# A Case of Avelumab-Resistant Upper Urothelial Carcinoma Responding to Pembrolizumab

**DOI:** 10.7759/cureus.77170

**Published:** 2025-01-08

**Authors:** Kotaro Yokota, Suguru Shirotake, Yu Miyama, Takayuki Takahashi, Yuta Umezawa, Masayuki Hagiwara, Go Kaneko, Masafumi Oyama

**Affiliations:** 1 Uro-Oncology, Saitama Medical University International Medical Center, Hidaka, JPN; 2 Pathology, Saitama Medical University International Medical Center, Hidaka, JPN

**Keywords:** cytotoxic chemotherapy, immune checkpoint inhibitor, metastatic urothelial carcinoma, upper urinary tract urothelial carcinoma, urinary tumor

## Abstract

In patients previously treated with immune checkpoint inhibitors (ICIs) for malignancies, the efficacy and safety of re-challenging with the same or another type of ICI remain unclear. We experienced a case in which a patient responded to pembrolizumab as a salvage treatment despite primary resistance to avelumab as a maintenance treatment for metastatic upper urinary tract urothelial carcinoma (mUTUC). A 69-year-old female presented with anemia and microscopic hematuria, diagnosed as mUTUC with lymph node metastases. After receiving avelumab maintenance therapy following platinum-based chemotherapy, there was no response at all. The previous chemotherapy was re-administered, but unfortunately, bone metastases were newly detected. We subsequently administered pembrolizumab to the patient, resulting in reductions in both the primary and metastatic tumors. Even if the patient has primary resistance to the anti-programmed death-ligand 1 (PD-L1) antibody avelumab, the anti-programmed cell death 1 (PD-1) antibody pembrolizumab, as an ICI rechallenge, could be a treatment option for a limited number of patients with metastatic urothelial carcinoma (mUC).

## Introduction

Upper urinary tract urothelial carcinoma (UTUC) originates from the urothelium of the renal pelvis and ureter. It accounts for 5-10% of all urothelial carcinomas, with a rare incidence of 2:100,000 in Western countries [1,2]. Metastatic upper urinary tract urothelial carcinoma (mUTUC) has a median overall survival of 12-15 months and is known to be a tumor with a poor prognosis [3].

In Japan, the standard first-line treatment for mUTUC involves chemotherapy, including platinum-based chemotherapy. Immune checkpoint inhibitors (ICIs) targeting programmed cell death 1 (PD-1) or programmed cell death ligand 1 (PD-L1) are used as second-line treatments: pembrolizumab as salvage treatment or avelumab as maintenance treatment following platinum-based chemotherapy [4,5]. The EV-301 clinical trial demonstrated the clinical benefit of enfortumab vedotin (EV) in all patients with advanced or metastatic urothelial carcinoma (UC) who had previously received platinum-based treatment and a PD-1 or PD-L1 inhibitor [6].

Here, we report a case of successful pembrolizumab treatment in a patient with mUTUC who was initially resistant to avelumab maintenance therapy.

## Case presentation

A 69-year-old Japanese woman presented at another hospital with anemia and microscopic hematuria. She was subsequently referred to our hospital for further evaluation and treatment. She had a history of an unruptured cerebral aneurysm and no notable family history. On examination, the patient weighed 50.9 kg, was 156.0 cm tall, and had a body surface area of 1.45 m^2^. The clinical findings revealed a hemoglobin level of 8.3 g/dL (reference range: 12 to 16 g/dL), a C-reactive protein level of 8.303 mg/dL (reference range: <0.3 mg/dL), and a mildly elevated creatinine level of 0.91 mg/dL (reference range 0.6-1.2 mg/dL); otherwise, the patient appeared normal (Table 1).

**Table 1:** Reference range

	Reference range
Hemoglobin	12-16 g/dL
C-reactive protein	<0.3 mg/dL
Creatinine	0.6-1.2 mg/dL

**Figure 1:**
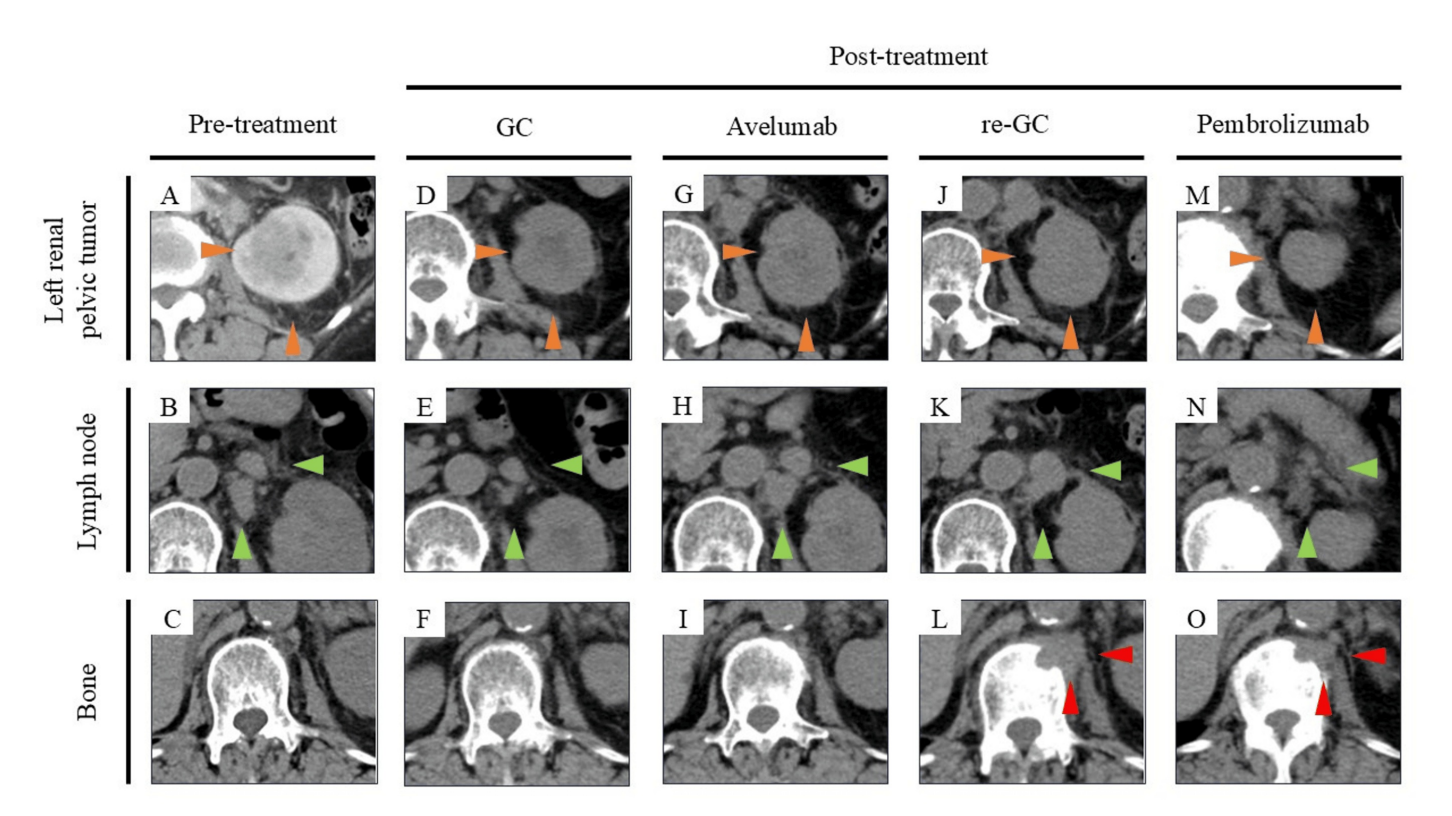
CT images of the left renal pelvic tumor, lymph node, and bone for every treatment Orange arrowheads show a left renal pelvic tumor. Green arrowheads show lymph nodes. Red arrowheads show bone metastasis. GC: gemcitabine and cisplatin combined chemotherapy, re-GC: rechallenged GC chemotherapy; CT: computed tomography

**Figure 2:**
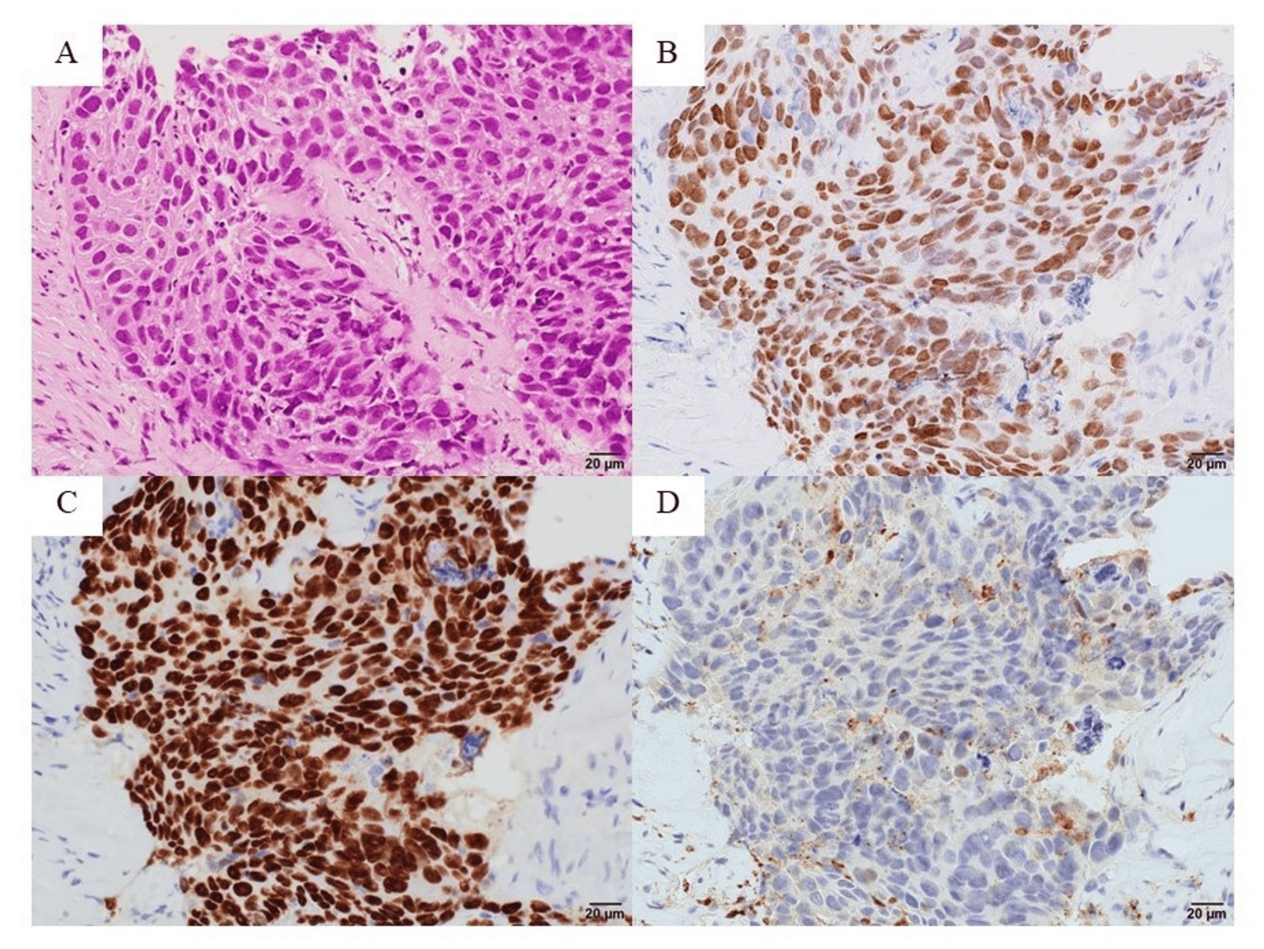
Pathological images of renal tumor tissue obtained from CT-guided biopsy at initial diagnosis A: hematoxylin and eosin staining. B: GATA3. C: p63. D: PAX8. Bar scale is 20 μm. CT: computed tomography

**Figure 3:**
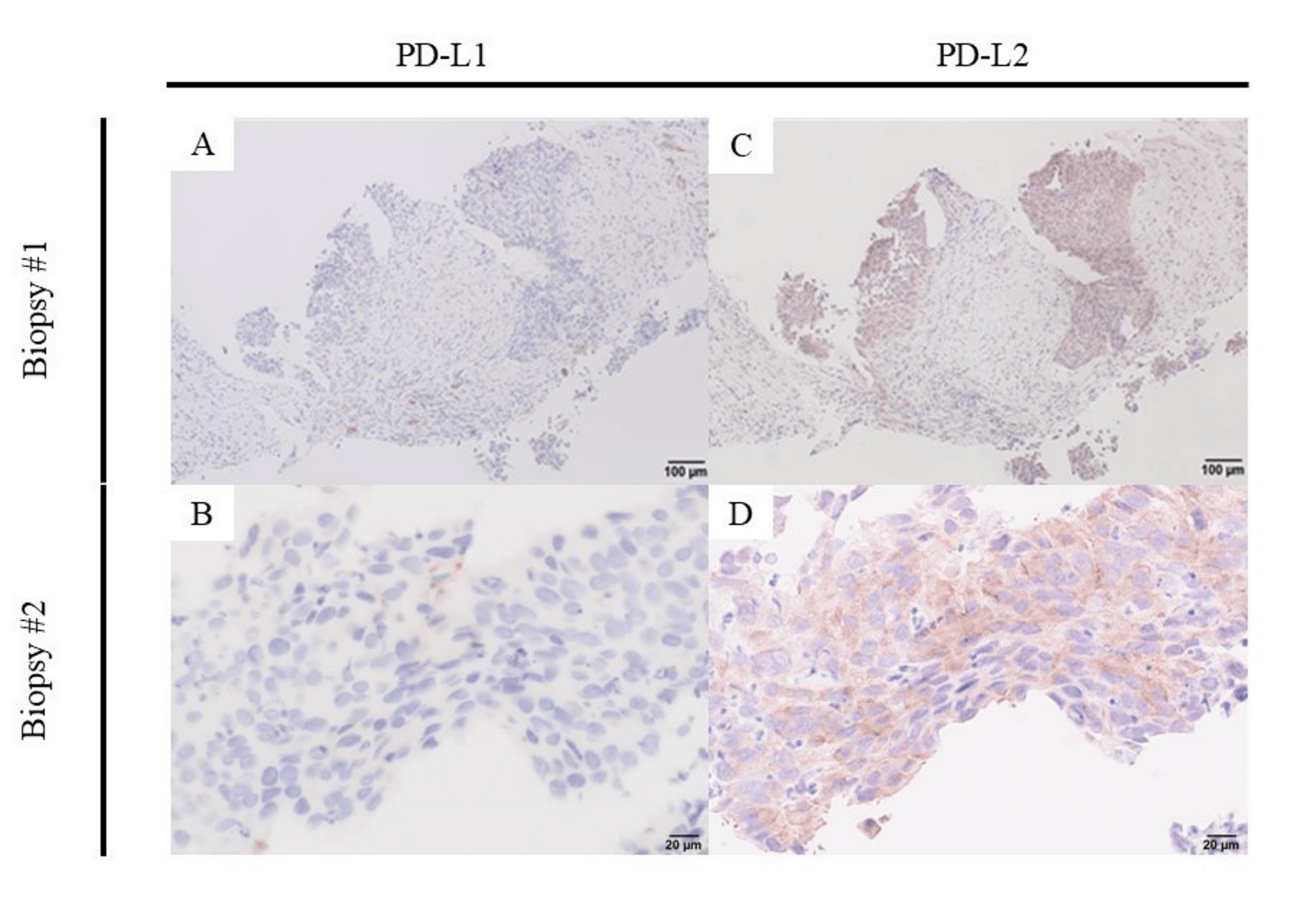
PD-L1 (A and B) and PD-L2 (C and D) expression in the renal tumor tissue at initial diagnosis Retrospectively performed immunohistochemical staining for PD-L1 and PD-L2. The bar scales are 100 μm for A and C and 20 μm for B and D. PD-L: programmed death-ligand

Urine cytology revealed atypical urothelial cells, while cystoscopy showed no abnormal findings. Computed tomography (CT) revealed a poorly enhanced left renal tumor and enlarged lymph nodes adjacent to the para-aorta (18 mm) and in the left renal pelvis (24 mm) (Figure 1A-1C). A CT-guided biopsy of the renal tumor, performed to assess the possibility of metastatic renal cancer and guide treatment planning, identified advanced invasive urothelial carcinoma, resulting in a diagnosis of mUTUC (cT3N2M0, Figure 2, Figure 3).

The patient underwent four cycles of GC therapy, consisting of gemcitabine (1000 mg/m² on days 1, 8, and 15) and cisplatin (70 mg/m² on day 2), administered every 28 days as salvage chemotherapy for mUTUC. The dosage of cisplatin was reduced based on decreased renal function. There were no significant adverse events. Following treatment, the renal tumor shrank, and the lymph nodes also decreased in size, with the nodes shrinking to 14 mm, resulting in a partial response (PR) (Figure 1D-1F) [7]. Although four cycles of maintenance therapy with avelumab (10 mg/kg) were administered every two weeks, the para-aortic lymph node enlarged to 20 mm and the left renal pelvis tumor grew to 22 mm, confirming progressive disease (PD) in the primary evaluation (Figure 1G-1I) [7]. There were no immune-related adverse events (irAEs) due to avelumab. Given the clinical benefits of the previous GC therapy, which effectively controlled the disease, and the patient's concerns about adverse events related to EV, an additional five cycles of GC therapy were administered. The primary tumor and lymph node metastases were stable (Figure 1J, 1K). However, multiple bone metastases were unfortunately identified in the 12th thoracic vertebra and the 1st lumbar vertebra (Figure 1L). The patient received pembrolizumab as a subsequent ICI rechallenge for two reasons. The first reason was that the efficacy and safety of EV for UTUC were initially unclear in the subgroup analysis of the EV301 trial, and the second was no occurrence of irAEs with the previous avelumab treatment.

**Figure 4:**
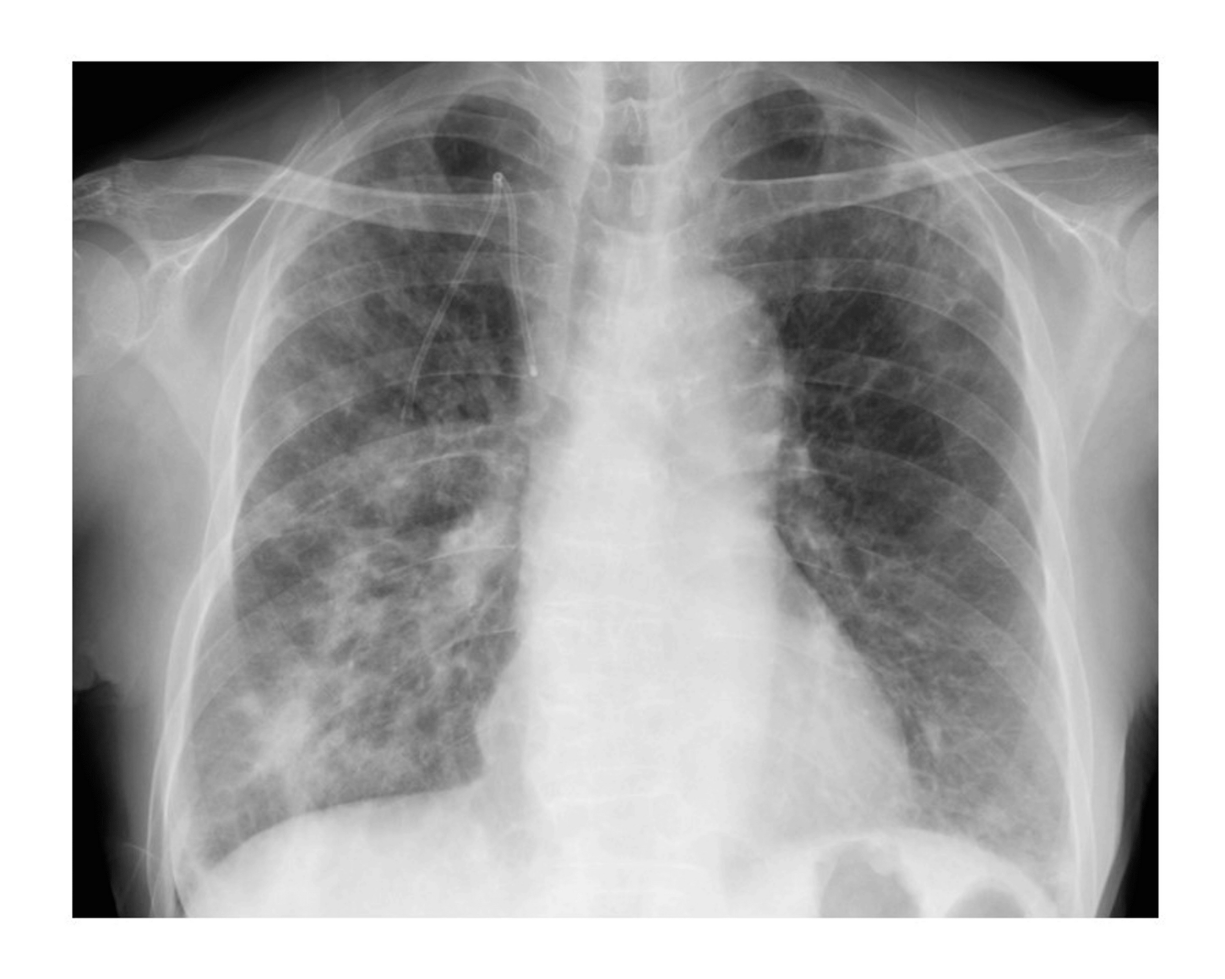
Chest X-ray after five cycles of pembrolizumab

Treatment with pembrolizumab (200 mg per dose once every three weeks) was initiated. After four cycles, a reduction in the size of the primary renal pelvic tumor, the para-aortic lymph node, the renal pelvis lymph node, and bone metastases was observed, leading to a response evaluation of PR (Figure 1M-1O) [7]. After five cycles of pembrolizumab, the patient complained of dyspnea in motion, and X-ray imaging revealed ground-glass opacities (Figure 4).

Blood tests indicated elevated Krebs von den Lungen-6 (KL-6) levels (2769 U/mL (normal range: <500 U/mL)), leading to a diagnosis of interstitial pneumonia as a grade 3 irAE according to CTCAE caused by pembrolizumab.

**Figure 5:**
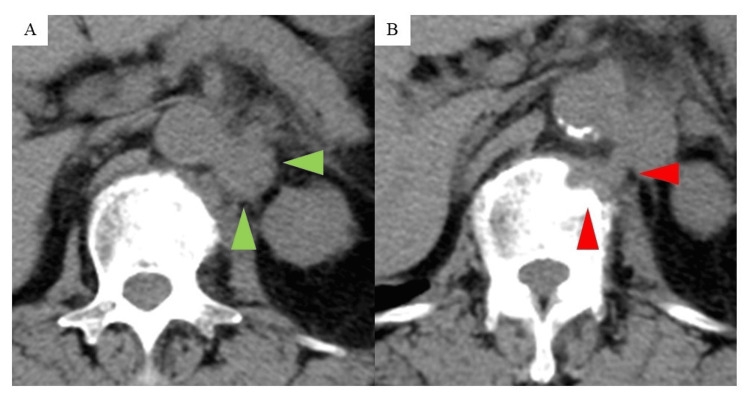
CT scan four months after discontinuing pembrolizumab treatment A: Green arrowheads show lymph nodes. B: Red arrowheads show bone metastasis. CT: computed tomography

Pembrolizumab was discontinued, and prednisolone (1 mg/kg (50 mg/body)) was administered. This improved respiratory symptoms and X-ray findings. KL-6 decreased to 1384 U/mL one month later and further reduced to 320 U/mL six months later. A CT scan four months after discontinuing pembrolizumab treatment revealed an increase in para-aortic lymph node metastasis and a worsening of thoracolumbar metastases, leading to the initiation of EV therapy (1.25 mg/kg) every 28 days (Figure 5).

## Discussion

Based on the results of the EV-301 trial, EV has been widely used as a standard of care in patients with metastatic urothelial carcinoma (mUC) who previously received platinum-based treatment and a PD-1 or PD-L1 inhibitor. On the other hand, the subgroup analysis in the EV-301 trial initially revealed that EV did not reduce the risk of death compared with chemotherapy in patients with UTUC [6].

Some observational studies of lung and kidney cancer have indicated that re-administration of the same or other types of ICI (re-ICI treatment) had some effect and that re-ICI treatment had almost no impact on the risk of irAE occurrence [8-10]. We experienced a case with mUTUC that had a favorable response and a severe irAE to anti-PD-1 antibody treatment (pembrolizumab) following anti-PD-L1 antibody maintenance treatment (avelumab). We discuss the successful response in this case, focusing on two points.

The first point is the significance of PD-L2 expression in malignancies. The difference between an anti-PD-L1 antibody and an anti-PD-1 antibody is the blocking target: only PD-L1 for avelumab (anti-PD-L1 antibody) and PD-L1 and PD-L2 for pembrolizumab (anti-PD-1 antibody). Although there are few reports on UC [11], PD-L2 is involved in clinical outcomes as a prognostic biomarker, and there is resistance to ICI in some cancers [12,13]. We also retrospectively investigated immunohistochemistry using biopsy specimens from the initial diagnosis, showing that tumor PD-L2 expression was more conspicuous than PD-L1 (Figure 3). Compared with PD-L1, the role of PD-L2 in the tumor microenvironment (TME) remains unclear. Further research is needed on the significance of tumor PD-L2 expression in TME.

Second, rechallenge chemotherapy before pembrolizumab may influence changes in the TME and the immunological effect due to the induction of immunogenic cell death [14] as well as the release of damage-associated molecular patterns to activate dendritic cells and induce tumor-specific cytotoxic T cells [11]. Unfortunately, a tissue biopsy was only performed at this case's initial diagnosis. While challenges remain, including physical invasiveness as well as mental and financial burdens, performing tumor and liquid biopsies before each treatment may allow us to assess TME, including PD-L2 expression, to guide the selection of optimal therapies. Recently, there have been some studies on liquid biopsy to assess the status of the systemic immune system and predict the therapeutic effect of ICI for UTUC, such as the neutrophil-to-lymphocyte ratio and fibroblast growth factor receptor 3 [12,13].

Based on this case, we expect to further elucidate the immune-related TME, including PD-L2 expression, explore safe biopsy methods before each treatment, and detect useful biomarkers for each malignancy.

## Conclusions

In this case, the temporary response to pembrolizumab in mUC, which did not respond to avelumab maintenance therapy, is significant. For patients who are either unable to use EV or show resistance to it, even if they have primary resistance to the anti-PD-L1 antibody avelumab, the anti-PD-1 antibody pembrolizumab as an ICI rechallenge could be a treatment option for patients with mUC. On the other hand, we must be very careful about the occurrence of various irAEs when selecting ICI re-treatment. In the future, reports on the efficacy and side effects of ICI re-treatment in mUC are expected.
